# Through the Lens of Expanded Scope of Practice: Training Requirements for Pharmacist-Prescribed Contraception Across the United States

**DOI:** 10.3390/pharmacy14030082

**Published:** 2026-06-02

**Authors:** Taylor Clark, Ioana Sandru, Samantha Kunkel, Sarah Lynch

**Affiliations:** 1 Department of Pharmacy Practice, Binghamton University School of Pharmacy and Pharmaceutical Sciences, Binghamton, NY 13905, USA; twarner1@binghamton.edu (T.C.); isandru1@binghamton.edu (I.S.); 2 Geisinger Health System, Danville, PA 17822, USA

**Keywords:** contraceptive prescribing, pharmacist prescribed contraceptives

## Abstract

Thirty-one U.S. states and the District of Columbia currently authorize pharmacists to prescribe hormonal contraception. Expansion of pharmacist scope of practice into areas such as contraceptive prescribing represents an extension beyond traditional baseline licensure authority. As a result, jurisdictions frequently require additional training to address competency, patient safety, and liability considerations. Because regulation of pharmacy practice occurs at the state level, training requirements vary across jurisdictions. State boards of pharmacy websites and publicly available regulatory materials were reviewed to identify training requirements for pharmacists authorized to prescribe hormonal contraception. Relevant statutes, regulations, and prescribing protocols were examined. Identified requirements were extracted and categorized into thematic groupings based on the level and type of training specified. Most jurisdictions require pharmacists to complete training beyond standard pharmacy education prior to prescribing hormonal contraception. Such requirements are characteristic of newly expanded scope-of-practice authorities, where regulators seek to verify competency and mitigate liability risk. As prescribing activities become more fully incorporated into pharmacy practice acts and professional education, separate training requirements may diminish over time.

## 1. Introduction

The ability to access and use contraceptive methods when desired is associated with many positive outcomes [1]. One of the major barriers to contraceptive access is the availability of clinicians and clinics that offer the full range of contraceptive options, a frequent issue in more rural areas of the United States [2]. Pharmacies are well positioned to serve as an additional access point, with pharmacists prescribing or furnishing contraceptives. This increased access opens clinician access, reduces patient barriers, and increases patient autonomy.

In the United States, thirty-one states and the District of Columbia (D.C.) specifically allow pharmacists to prescribe or furnish self-administered hormonal contraceptives [3,4]. Various terms are used in regulations that define this practice, including “prescribe,” “dispense,” and “furnish.” For simplification, this paper will use the word “prescribing” to encompass all of these. Legislative restrictions vary among states including minimum age of service, eligible dosage forms, and pharmacist training requirements.

### Context

In the United States, pharmacists complete a Doctor of Pharmacy (PharmD) degree from a program accredited by the Accreditation Council for Pharmacy Education (ACPE) and must pass national and state licensure examinations to practice. Pharmacy curricula generally include core instruction in contraception and patient self-care; however, the depth of contraceptive prescribing training and experiential exposure may differ between institutions. Many states also require pharmacists to complete continuing education for license renewal, although the specific requirements vary.

While ACPE establishes national educational standards, individual states regulate pharmacy practice through their respective state boards of pharmacy, resulting in variation in pharmacist scope of practice and prescribing authority across states. States allow certain activities to fall within standard licensing scope, but other activities may require additional regulatory mechanisms. This includes the use of non-patient specific protocols or collaborative practice agreements. Non-patient specific protocols may be issued by a state health officer or supervising prescriber while collaborative practice agreements (CPA) typically involve formal arrangements between a pharmacist and a physician or other authorized prescriber that allows pharmacists to perform specified patient care functions. Some states have also adopted category-specific prescribing authority in which pharmacists may independently prescribe medications for designated conditions after completing required training or following standardized clinical algorithms.

Traditionally, when states first expand pharmacist scope of practice to encompass a service historically provided by only physicians or advanced practice practitioners, proof of competency or training is often required [5]. This includes engaging in activities that extend beyond traditional dispensing functions such as expanded prescriptive authority, diagnostic assessment, or protocol-driven clinical decision-making. The additional training required to participate in these expanded roles act as a regulatory safeguard. Extra training may be added to verify competency for new authority, liability, and patient safety. Training requirements are often incorporated for facilitating legislation adoption when expanding pharmacist authority. A clinical activity becomes part of routine pharmacist scope of practice when it is incorporated into standard licensure authority and does not require separate statutory authorization, certification, or institutional privilege [6].

States have authority to define training and certification requirements for pharmacists participating in these activities. Training requirements may be general, or a specific program may be required. For example, the American Pharmacists Association Pharmacy-Based Immunization Certificate Training Program is considered a prerequisite for pharmacist immunizers in many states [7]. States can build in additional competency requirements by requiring specific continuing education during each registration cycle for all pharmacists or those providing the specific service.

Some states that authorize pharmacist hormonal contraceptive prescriptive authority include contraceptive prescribing as a standard element of pharmacist scope, which means that pharmacists who learned about hormonal contraceptive prescribing within their pharmacy school curriculum can prescribe upon licensure. Other states require additional post-graduate training or certification programs for pharmacists to prescribe contraceptives in the United States.

This paper summarizes the training requirements by state for pharmacist prescribed hormonal contraceptives.

## 2. Materials and Methods

State boards of pharmacy websites and publicly available regulatory materials were systematically reviewed to collect information on training requirements for pharmacists authorized to prescribe contraceptives, for those states that allow pharmacist prescribing. Initial identification of states with pharmacist contraceptive prescribing authority was conducted using publicly available summaries from national organizations, including National Alliance of State Pharmacy Associations and KFF. State-specific statutes, regulations, protocols, and board of pharmacy guidance documents were then reviewed directly from official state government and regulatory websites to verify pharmacist prescribing authority, characterize the mechanism of authorization, and confirm the status of remaining states without identified authority. Jurisdictions were included if they had enacted legislation specifically addressing pharmacist-prescribed or pharmacist-dispensed contraceptives. While several states authorize prescribing under a general state practice act or through non-specific collaborative practice agreements (CPAs), these pathways were excluded from further categorization due to their lack of specificity to hormonal contraceptive prescribing. States with proposed legislation were also excluded.

The data collection was carried out from January through March 2026. For each jurisdiction, applicable statutes, regulations, board guidance documents, and prescribing protocols were examined. Extracted training and documentation requirements were compared across jurisdictions and categorized into thematic categories according to type of training specified. Thematic categories of training requirements were defined by all four authors. Each state was independently categorized by all four authors based on the predefined study criteria. Following independent review, categorizations were compared across authors to identify discrepancies. Disagreements were discussed collectively during consensus meetings, with authors reviewing the relevant statutory and regulatory language together until agreement was reached on the final classification for each state. Frequencies and percentages were described using descriptive statistics.

## 3. Results

Thirty-one states plus the District of Columbia met inclusion criteria. The authors identified that training and documentation requirements fell into eight separate categories, defined in Table 1. Each jurisdiction had at least one applicable category, with many states meeting multiple criteria. Each jurisdiction’s requirements are described in Table 2.

Most jurisdictions require some form of additional training for pharmacists to prescribe hormonal contraceptives (Table 3). Only one state, Utah, explicitly stated that pharmacy interns were also eligible to prescribe hormonal contraceptives [35]. The most common training requirement was completion of a state-specified training program, reported in 16 states. Other common training pathways included any ACPE accredited training program, and training programs meeting defined statutory or regulatory requirements. Considerable variability was observed in both the type and rigor of the training requirements across jurisdictions.

Nine states authorize training within an accredited school of pharmacy curriculum, effectively establishing this service as part of standard scope of practice upon graduation.

Only five states explicitly require that documentation is submitted to the state prior to offering the service; the majority seem to entrust pharmacists with their own training and recordkeeping. Continuing education requirements specific to contraceptive prescribing were identified in eight states, with variability in frequency and hour requirements.

Idaho has no additional training requirements. Vermont regulations are less explicit, stating only that it requires “training and education sufficient to perform duties.” While jurisdictions that offer general prescribing under a CPA were excluded, Connecticut was included in the data as it represents a hybrid model, permitting prescribing under a CPA while also providing a pathway requiring completion of an approved training program.

## 4. Discussion

Pharmacist-prescribed contraceptives represent a relatively recent expansion of scope of practice, first authorized in California in 2013 [40]. Contraceptive prescribing is following a broader pattern in which states introduce additional training or credentialing requirements when expanding pharmacist scope. Training requirements overall vary widely, ranging from no additional training to completing specific state-approved training courses. Some jurisdictions allow pharmacy school curricula to satisfy training requirements, highlighting an opportunity for academic programs to better prepare graduates for expanded scope. Most do not require submission of training documentation prior to prescribing, instead requiring pharmacists to maintain proof if requested, which helps reduce administrative barriers.

The authors were able to classify regulatory approaches into eight distinct categories, but the absence of a standardized national framework has resulted in variability beyond these eight categories. For example, in Utah, pharmacy interns are specifically permitted to prescribe hormonal contraception, expanding authority beyond licensed pharmacists. While this may increase service capacity and provide experiential learning opportunities, it also introduces considerations related to supervision, competency, and liability [35]. Utah also requires both pharmacists and interns to re-enroll every two years, creating an administrative burden that may risk lapses in authorization. In contrast, Vermont requires that pharmacists complete training “sufficient to perform the duties involved,” a vague standard that differs from more common state- or ACPE-specified training requirements [36].

The authors elected to exclude collaborative practice agreements, focusing instead on legislation that explicitly authorizes contraceptive prescribing. While CPAs represent a legitimate pathway for prescribing in states where such authority is not directly incorporated into the pharmacy practice act, it is worth noting that they frequently entail additional credentialing or training requirements.

While contraceptive prescribing represents one of the first examples of widespread independent prescribing scope in the United States, countries such as Canada, New Zealand, the United Kingdom, and Australia have been expanding pharmacist prescribing since the early 2000s, extending beyond contraception-specific services to broader medication management and primary care functions. Canada’s framework most closely resembles that of the United States because prescribing authority is regulated at the provincial rather than national level. In all Canadian provinces, pharmacists receive baseline prescribing authority for “minor ailments” upon licensure, while more advanced or independent prescribing authority may require additional credentialing, competency review, or practice experience [41,42]. In contrast, pharmacist prescribing models in New Zealand, the United Kingdom, and many Australian programs generally require pharmacists to complete additional postgraduate prescribing education, supervised clinical training, and/or advanced credentialing before independently prescribing medications [43]. In the United Kingdom and New Zealand, pharmacist prescribers typically practice within formally recognized prescribing scopes tied to competency standards and collaborative care models.

In surveys related to pharmacist prescribed contraceptives, training is frequently cited as both a facilitator and a barrier: pharmacists frequently report supporting structured training, including a preference for live programs running three to four hours long [44]. However, it is also reasonable to expect that additional requirements will limit participation due to time, cost, and accessibility challenges.

Beyond training, states differ in protocols, patient eligibility criteria, and product scope [45]. Many require use of screening tools, either state-specific or based on evidence-based resources such as those from the Centers for Disease Control and Prevention. Thirteen states impose minimum age requirements, and sixteen states allow dispensing of extended contraceptive supplies, despite evidence supporting improved adherence with longer durations. While all states providing this service permit prescribing of self-administered methods (e.g., oral contraceptives, patches, vaginal rings), some also allow pharmacist administration of depot medroxyprogesterone.

Although pharmacist prescribing is often cited as a method to improve patient access, inconsistencies in how it is authorized and operationalized, beyond just training, may also limit its intended impact. For example, Colorado and Arkansas have eligibility requirements dependent on seeing a healthcare provider within timeframes ranging from six months to three years [45]. Such requirements may perpetuate physician-dependent access pathways and diminish the accessibility advantages of pharmacist prescribing, particularly for individuals seeking to avoid delays associated with traditional care models.

Despite expanded authority, pharmacist prescribed contraceptive services remain underutilized. Pharmacists account for a small proportion of total contraceptive prescriptions, and individual prescribing volume is low [46,47]. Of twenty states reviewed in 2022, pharmacists had only prescribed a small percentage of total hormonal contraceptive prescriptions written, with the highest state average having pharmacists writing 56 per 10,000 total contraceptive prescriptions. The study also found that the average pharmacist who participates in prescribing had written less than 10 prescriptions. Low uptake by pharmacists may also be the result of nonexistent or poor reimbursement for services. It may also be partially due to a diffusion-innovation effect, which predicts that uptake of new services accelerates when service visibility increases, participants observe successful use, and social normalization occurs [48]. This may describe the current self-perpetuating cycle in which pharmacists do not routinely offer the service, resulting in limited availability, which reduces patient awareness and utilization, and in turn further discourages pharmacists from offering the service.

The variability found here parallels earlier expansions in pharmacist scope of practice, such as immunization authority, which initially involved inconsistent training, age restrictions, product limitations, and insurance coverage [6,7]. Over time, however, these elements have become more standardized, and immunization services are now considered an expected component of community pharmacy practice. Precedents like this suggest that pharmacist-prescribed contraception may similarly evolve toward greater harmonization. An important consideration is that harmonization should not be equated with the establishment of a single training program as the universal standard. Such an approach may limit flexibility, reducing states’ ability to adapt if the program fails to maintain rigorous standards or does not incorporate evolving clinical evidence in a timely manner.

One answer to these challenges is for greater adoption of “standard of care” regulatory models, which allows greater flexibility for pharmacists to perform a broad range of services that align with their training and experience [43]. Public health needs and medical advancements evolve rapidly, and the current regulatory model in pharmacy requires pharmacists to petition for legal authority to explicitly offer new services. A standard of care model would allow for easier adaptation for the profession, and could in turn simplify the process for pharmacists who relocate or practice across state lines.

The standard of care regulatory model describes a pathway to reduce the need for highly prescriptive, state-specific training requirements for pharmacist-prescribed contraception [49]. Rather than mandating detailed procedural training tied to rigid protocols, the standard of care model aligns pharmacist authority with broader healthcare standards, allowing pharmacists to exercise clinical judgment consistent with accepted medical practice. In the context of birth control prescribing, this approach can shift the emphasis from completing additional requirements, such as variable state-mandated certification programs, to demonstrating competence through professional education, licensure, and adherence to evidence-based guidelines. This may decrease regulatory burden and redundancy in training, facilitating more consistent implementation across states.

There were several limitations to this study. Data sources were primarily publicly available materials. Contraceptive prescribing represents a rapidly evolving regulatory landscape, and publicly available materials are not always updated in a timely manner. Additionally, states that allow for contraceptive prescribing via collaborative practice agreement may have been unintentionally excluded. Collaborative practice agreements can be complex and may include numerous caveats, making them difficult to clearly categorize; therefore, the authors limited this study to states that explicitly authorize contraceptive prescribing.

## 5. Conclusions

The landscape of pharmacist-prescribed hormonal contraception across the United States represents an expanded scope of pharmacist practice, which is typically accompanied by specific regulatory requirements. Our review of the states authorizing this practice reveals a diverse and sometimes fragmented approach to ensuring pharmacist competency through training. Comprehensive summaries of training requirements for other pharmacist scope-of-practice expansions are limited; however, available evidence and regulatory review suggest substantial variability across states. Training requirements for activities such as administering long-acting injectable medications, performing point-of-care testing, and prescribing or managing therapy for chronic disease states differ considerably with respect to required training and eligibility. While a minority of states have integrated contraceptive prescribing into standard pharmacist scope, the majority continue to mandate additional post-graduate training, such as state-specified programs, ACPE-accredited courses, or training that meets defined statutory criteria. These supplementary training requirements are consistent with the regulatory pattern observed when new clinical activities are first introduced into the pharmacy profession, often presented as mechanisms for verifying competency and mitigating potential patient safety and liability risks. As this practice matures, and as pharmacy school curricula increasingly incorporate comprehensive training on hormonal contraception prescribing, it is anticipated that the need for separate, mandatory post-licensure training and documentation submission may lessen. Adoption of a standard of care model of practice, already underway in several states, could potentially speed up this process for both contraceptive prescribing and for other clinical services. Ultimately, aligning training requirements with the core goal of increasing patient access, while maintaining rigorous standards of care, remains critical for the successful and widespread adoption of pharmacist-prescribed contraception.

## Figures and Tables

**Table 1:** Training Requirement Categories.

Training Requirement Category	Definition
No additional training requirement	Regulations do not define specific training requirements or specifically state that no additional training is required.
Authorization granted under the state practice act or through a collaborative practice agreement	Authority to prescribe contraceptives is included as part of pharmacist standard scope or through a collaborative practice agreement.
State-specified training program(s)	The regulations define one or more specific training programs or require a program that has been approved by the state board of pharmacy
Training programs meeting defined statutory or regulatory criteria	The regulations define specific topics that must be covered in the training program, but do not define a specific training program.
Any American College of Pharmaceutical Education (ACPE) accredited training program	The regulations define that the training program must be ACPE accredited and on the topic of hormonal contraception.
Training incorporated within an accredited pharmacy school curriculum	The regulations state that training can occur as part of an accredited pharmacy school’s curriculum.
Must submit documentation of training completion to the state board before prescribing	The regulations require pharmacists to submit some type of documentation to the state board of pharmacy before participating in prescribing.
Completion of continuing education (CE)	Pharmacists are required to complete topical continuing education during registration cycles.
Other	Training does not qualify as any of the above

**Table 2:** Training Requirements for Pharmacist Prescribing of Hormonal Contraceptives by State.

State/Jurisdiction *n* = 32	Training Required	Type of Training	CE Requirement	Board Approval Requirement
Arizona [8]	Yes	State Specified Training Program(s) Completion of CE	3 h	No
Arkansas [9]	Yes	State Specified Training Program(s)	CE Encouraged	No
California [10]	Yes	Training incorporated within an accredited pharmacy school curriculum Completion of CE	Minimum 1 h of CE before prescribing	No
Colorado [11]	Yes	Any ACPE-accredited training program	Not Specified	No
Connecticut [12]	Yes	Authorization granted under the state practice act or through CPA Any ACPE-accredited training program	Not Specified	No
Delaware [13]	Yes	Training incorporated within an accredited pharmacy school curriculum Any ACPE-accredited training program	Not Specified	No
District of Columbia [14]	Yes	Training incorporated within an accredited pharmacy school curriculum Training program meeting defined statutory or regulatory criteria	Not Specified	No
Hawaii [15]	Yes	State Specified Training Program(s) Completion of CE	Yes	Yes, submit proof of completion of CE
Idaho [16]	No	No additional training required	No	No
Illinois [17]	Yes	Any ACPE-accredited training program	Not Specified	No
Indiana [18]	Yes	State Specified Training Program(s)	Not Specified	No
Kentucky [19]	Yes (norgestrel 0.075 mg only)	State Specified Training Program(s)	Not Specified	No
Maine [20]	Yes	Training program meeting defined statutory or regulatory criteria	Not Specified	Yes, submit documentation (application and fee) of training completion
Maryland [21]	Yes	Training incorporated within an accredited pharmacy school curriculum State Specified Training Program(s) Completion of CE	1 h before license renewal date	Yes, at least 15 days before prescribing must submit Training Notification Form
Massachusetts [22]	Yes	Training program meeting defined statutory or regulatory criteria	Encouraged	No
Michigan [23]	Yes	State Specified Training Program(s)	Not Specified	No
Minnesota [24]	Yes	Training incorporated within an accredited pharmacy school curriculum Any ACPE-accredited training program Training program meeting defined statutory or regulatory criteria	Not Specified	No
Nevada [25]	Yes	Training incorporated within an accredited pharmacy school curriculum Training program meeting defined statutory or regulatory criteria	Not Specified	Yes, documentation of intent to dispense must be submitted
New Hampshire [26]	Yes	State Specified Training Program(s) Completion of CE	Yes, biennial	No
New Jersey [27]	Yes	Training incorporated within an accredited pharmacy school curriculum State Specified Training Program(s)	Not Specified	Yes, must submit certificate of completion and written affirmation before prescribing
New Mexico [28]	Yes	State Specified Training Program(s) Completion of CE	Minimum of 0.2 CEU live biennial	No
New York [29]	Yes	Training program meeting defined statutory or regulatory criteria	Not Specified	No
North Carolina [30]	Yes	State Specified Training Program(s)	Not Specified	No
Oregon [31]	Yes	Training program meeting defined statutory or regulatory criteria	Not Specified	No
Rhode Island [32]	Yes	State Specified Training Program(s)	Not Specified	No
South Carolina [33]	Yes	Training incorporated within an accredited pharmacy school curriculum Training program meeting defined statutory or regulatory criteria Completion of CE	Minimum 1 h of ACPE or continuing medical education	No
Tennessee [34]	Yes	Training incorporated within an accredited pharmacy school curriculum State Specified Training Program	Encouraged	No
Utah [35]	Yes	State Specified Training Program(s) Completion of CE	Yes, 2 h each renewal period	No
Vermont [36]	Yes	Other “have training and education sufficient to perform duties”	Not Specified	No
Virginia [37]	Yes	Any ACPE-accredited training program	Not Specified	No
Washington [38]	Yes	State Specified Training Program(s)	Not Specified	No
West Virginia [39]	Yes	State Specified Training Program(s)	Not Specified	No

**Table 3:** Summary of Training Requirements for Pharmacist Prescribing of Hormonal Contraceptives.

Training Requirement Category	Number of Jurisdictions ^1^ *n* = 32
State-specified training program(s)	16, (50.0%)
Training incorporated within an accredited pharmacy school curriculum	9, (28.1%)
Completion of continuing education	8, (25.0%)
Training programs meeting defined statutory or regulatory criteria	8, (25.0%)
Any ACPE-accredited training program	6, (18.8%)
Documentation of training completion to the state board required	5, (15.6%)
No additional training requirements	1, (3.1%)
Authorization granted under the state practice act or through a collaborative practice agreement	1, (3.1%)
Other	1, (3.1%)

^1^ Percentages do not sum to 100%, as multiple categories could apply to a single state.

## Data Availability

The original data presented in the study are openly available publicly (see references [8,9,10,11,12,13,14,15,16,17,18,19,20,21,22,23,24,25,26,27,28,29,30,31,32,33,34,35,36,37,38,39] below).

## Abbreviations

The following abbreviations are used in this manuscript:

CPA	Collaborative Practice Agreement
ACPE	American College of Pharmaceutical Education
CE	Continuing Education
